# Supplementary material for: An evolutionarily conserved iron-sulfur cluster underlies redox sensory function of the Chloroplast Sensor Kinase
Source: Commun Biol. 2020 Jan 8;3:13. doi: 10.1038/s42003-019-0728-4 (PMC6949291; doi:10.1038/s42003-019-0728-4)
Supplement: Supplementary file 1 — Supplementary Information [file 42003_2019_728_MOESM1_ESM.pdf]

Supplementary Figures

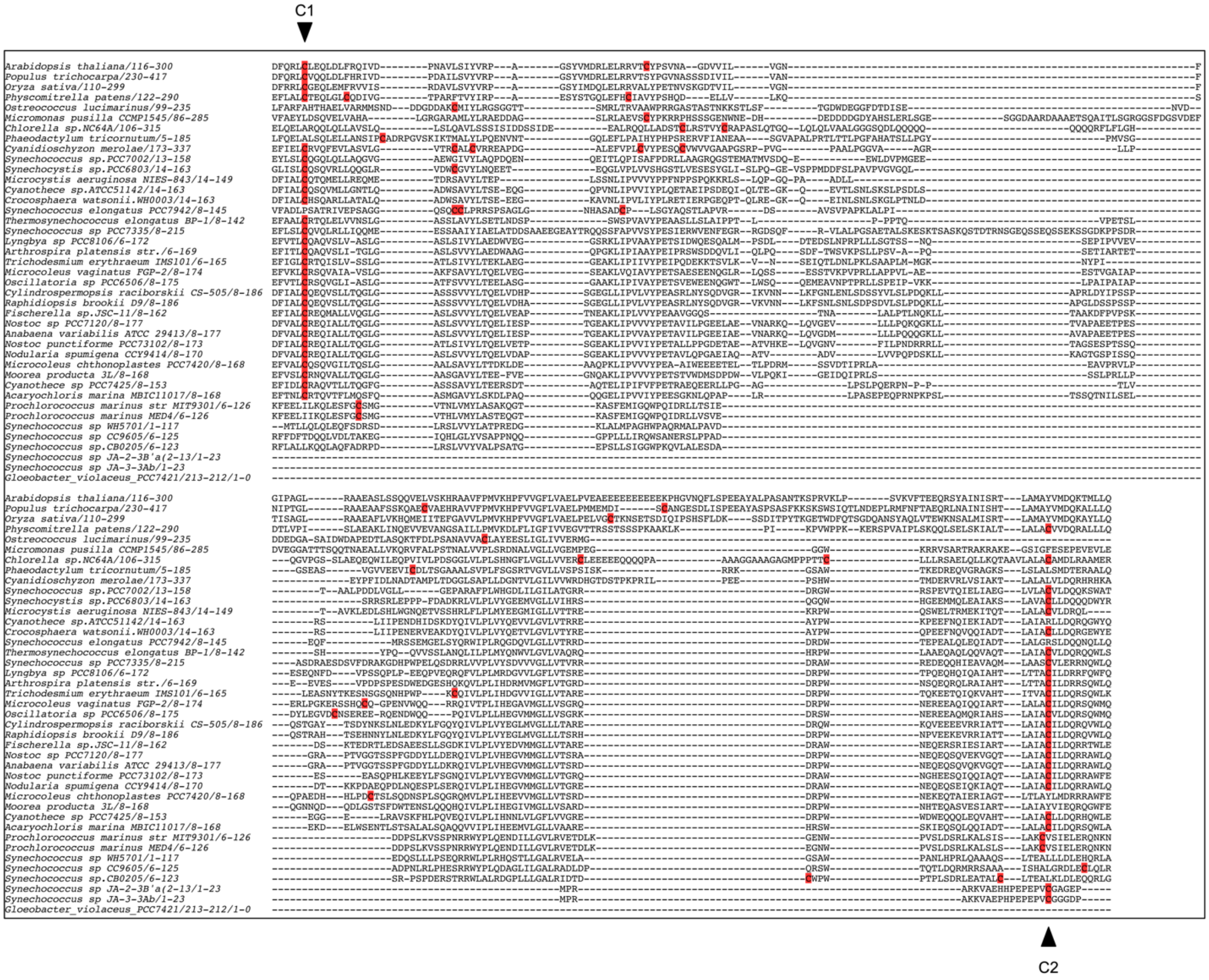

Supplementary Figure 1. Conserved cysteine residues within the GAF domain of CSK. A multiple sequence alignment of plant, algal and cyanobacterial CSK orthologues showing the GAF domain. The cysteine residues are highlighted in red and the two conserved putative Fe-S-binding cysteines are marked by an arrowhead.

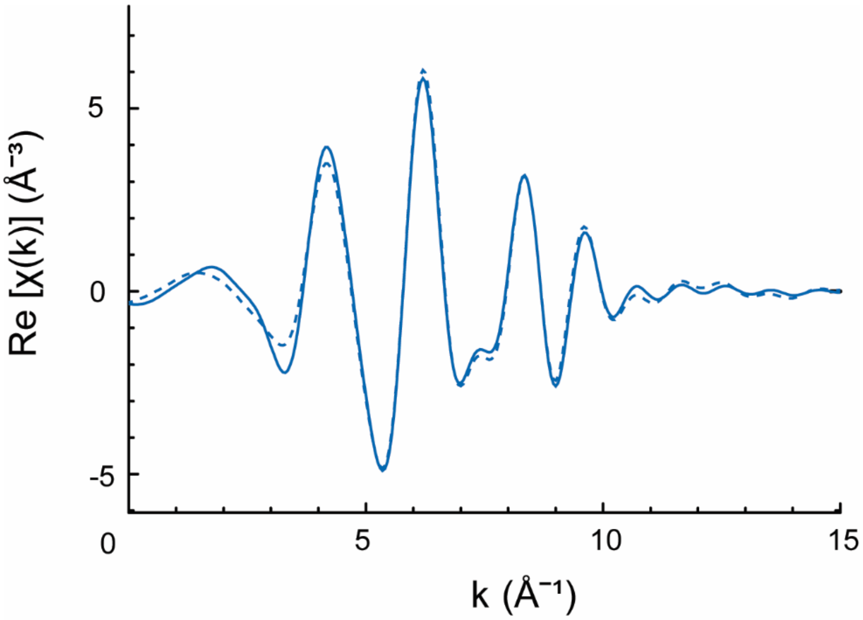

Supplementary Figure 2. Fitting of Syn-CSK protein (solid line) in q space with fit 5 (dashed line) from Table 1.

a

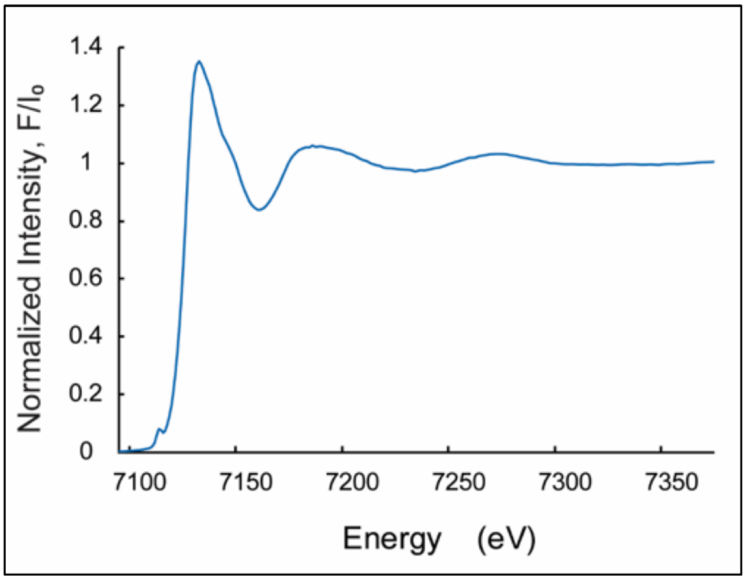

b

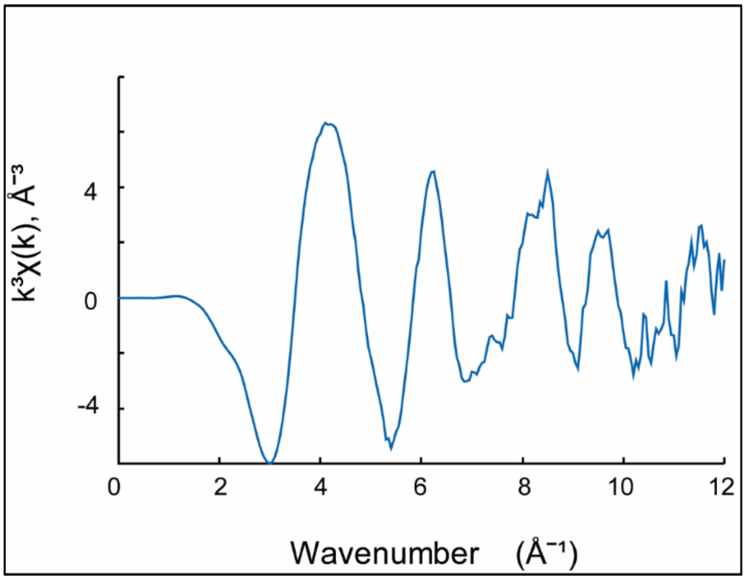

Supplementary Figure 3. a Extended normalized Fe K-edge XANES spectra of Syn-CSK protein. **b** Raw k-space EXAFS of Syn-CSK protein.

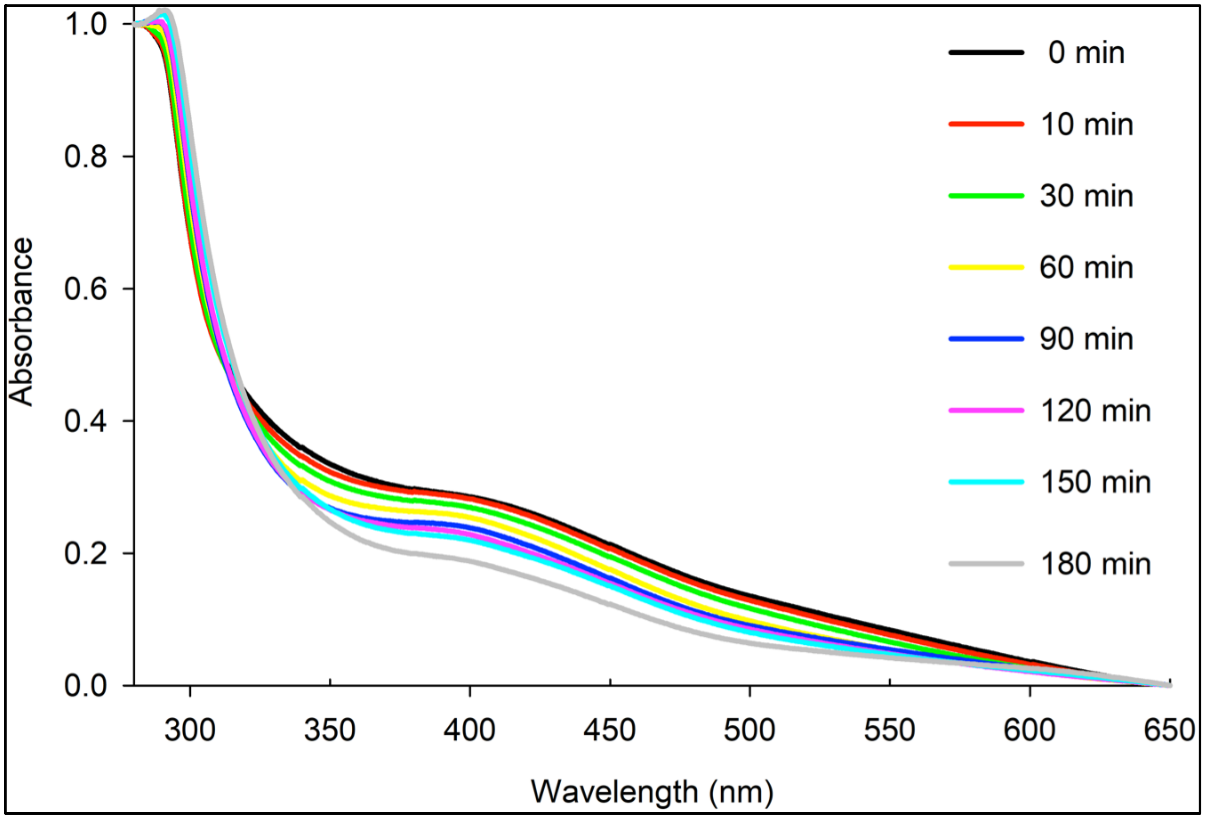

Supplementary Figure 4. The Fe-S cluster of CSK is oxygen-tolerant. The UV-Vis absorbance spectrum of Syn-CSK immediately after transfer from anaerobic (reconstitution) to aerobic conditions (time zero). The absorbance starts to decrease gradually upon exposure to air. After 180 minutes of incubation, the absorbance at 410 nm has decreased only by 36 % relative to the initial value at time zero, implying that the CSK cluster is fairly stable at room temperature (22 ºC).

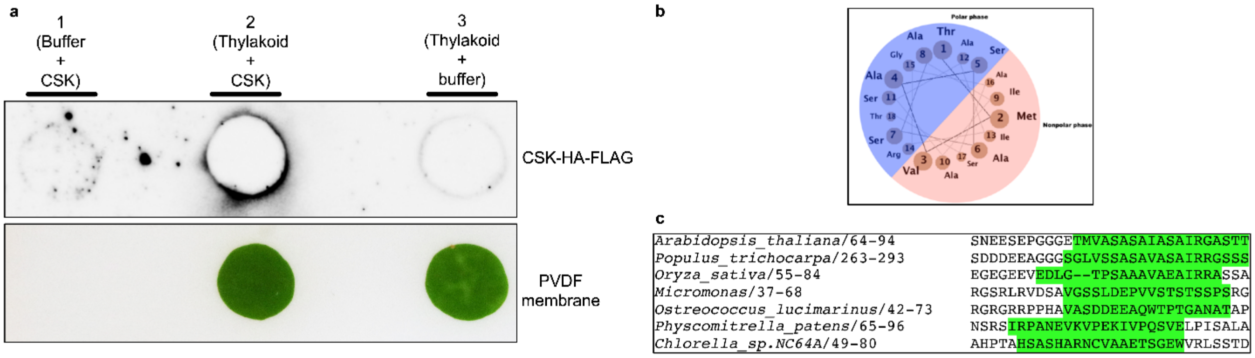

Supplementary Figure 5. Thylakoid membrane interaction of CSK. **a** A dot immunoblot showing thylakoid membrane interaction of CSK. The dot blot labeled 1 represents a negative control, which demonstrates that the CSK protein does not associate non-specifically with the PVDF membrane in the absence of thylakoid. The dot blot marked as 3 is another control that rules out non-specific binding of the primary or secondary antibody to the thylakoid membrane in the absence of the CSK protein. The rim of the dot blot, shown in 2, has more immunoreactive signal, likely due to poor binding of the primary antibody in the chlorophyll dense middle region. **b** Helical wheel modeling of the predicted *Arabidopsis* CSK amphipathic helix. Hydrophobic residues are highlighted in red and hydrophilic residues are highlighted in blue. **c** A multiple sequence alignment of the predicted amphipathic helix of green algal and plant CSK. The amino acid sequence forming the amphipathic helix is shaded in green. This sequence motif is found in a region immediately adjacent and N-terminal to the redox sensory GAF domain. The multiple sequence alignment was generated with the clustalOmega program and edited with Jalview.

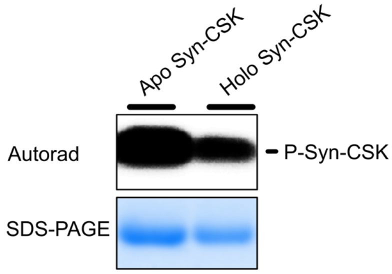

Supplementary Figure 6. Kinase activity of Apo and holo Syn-CSK.

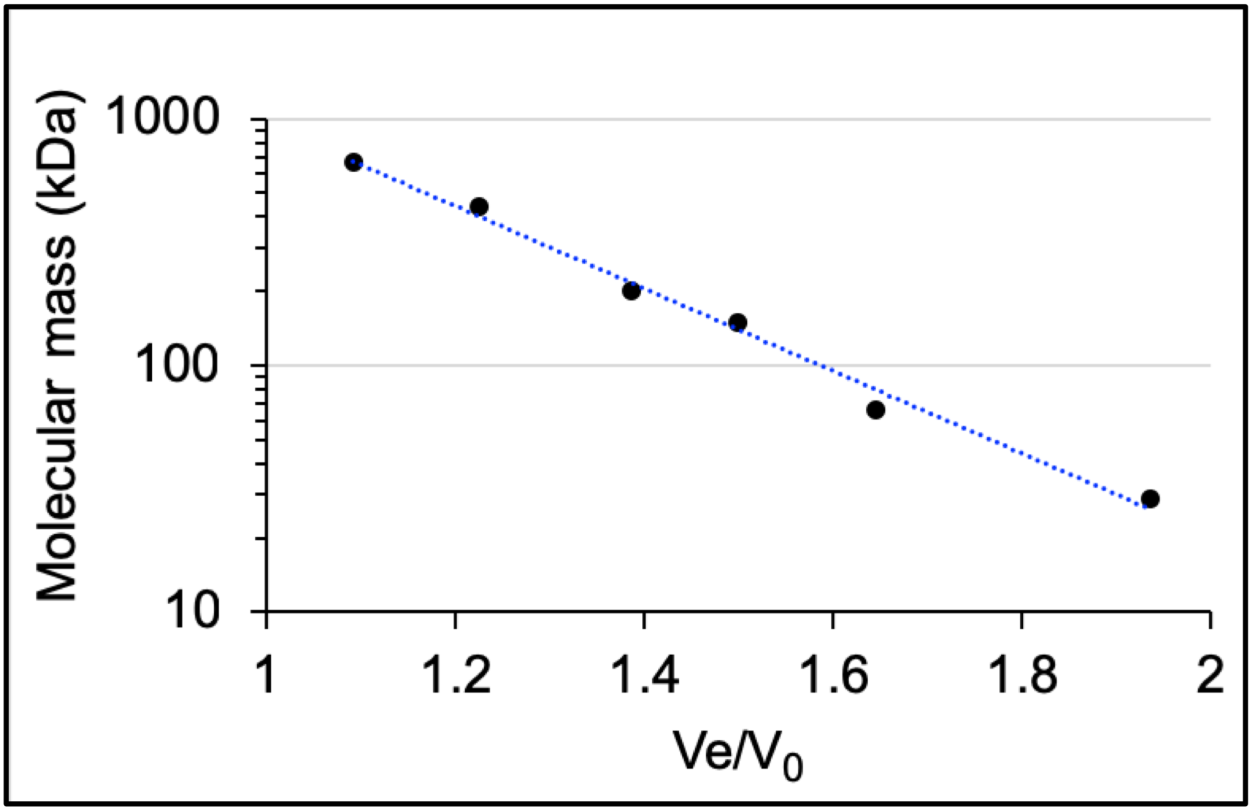

Supplementary Figure 7. Calibration curves of the Superdex 200 column using protein molecular weight standards: Calibration curve was obtained using standard proteins of known molecular mass: thyroglobulin (669 kDa), apoferritin (443 kDa), β-amylase (200 kDa), alcohol dehydrogenase (150 kDa), bovine serum albumin (66 kDa) and carbonic anhydrase (29 kDa). Blue dextran (2000 kDa) was used to determine the void volume (Vo). Ve is the effluent volume. On the y-axis the base ten logarithm of the protein molecular mass is shown and on the x-axis, Ve/V_0.

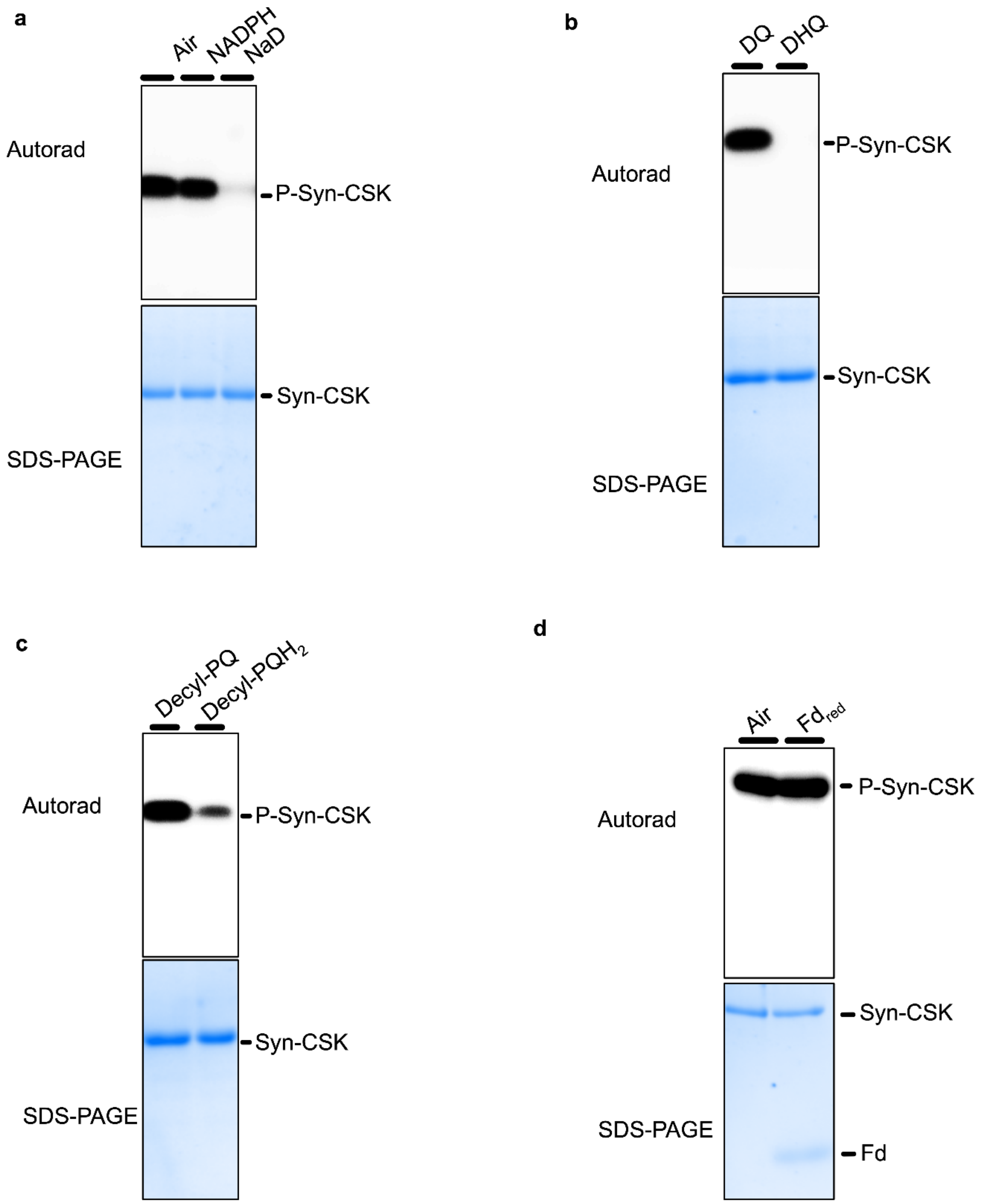

Supplementary Figure 8. The full uncropped autoradiographs of CSK autokinase activity and the corresponding stained SDS-PAGE gel presented in Fig. 6.

Supplementary Tables

	Fe^{2+} (mole protein)$^{-1}$	S^{2-} (mole protein)$^{-1}$
**Syn-CSK^{Wt}**	3.0 ± 0.07	4.0 ± 0.85
**Syn-CSK^{C19S}**	1.0 ± 0.03	1.9 ± 0.22
**Syn-CSK^{C35S}**	2.2 ± 0.11	4.0 ± 0.19
**Syn-CSK^{C153S}**	3.1 ± 0.02	2.3 ± 0.19
**Syn-CSK$^{C19S/C35S/C153S}$**	1.4 ± 0.04	2.7 ± 0.43
**Ara-CSK^{Wt}**	2.8 ± 0.08	3.7 ± 0.46

Supplementary Table 1: Analysis of CSK for iron and labile sulfide

± Standard error (n = 3-4)

**Protein name**	**TAIR ID**	**Light conditions**	**Phosphopeptide sequence**	**m/z**	**Spectral count**	**Score/q-value**
Chloroplast Sensor Kinase	AT1G67840	Far-red	AAEASLSSQQVELVSK	1726.83	2	0.034
		Orange	-	-	n.d.	-

Supplementary Table 2: Phosphorylation in *Arabidopsis* CSK. The phosphorylation site is indicated in red.

Purpose	Clone or Gene ID	Primer name	Sequence 5’ to 3’
**Cloning**	Syn-CSK-His$_6$ (cloned in pET-21b)	Syn-CSK_F	GCGCGCcatatgGCCGGTTCCATCTCA
		Syn-CSK_R	GCGCGCctcgagCACTTGTTCTCCAGAGCG
	Phaeo-His$_6$-MBP-CSK (cloned in pETG-41A)	Phaeo-CSK_F	GCGGcatatgGTAACGGAAAACATCAGGT
		Phaeo -CSK_R	GCGGctcgagTCACCAGTGTTTTCGCAATG
	Ara-CSKF-His6 (cloned in pET-21b)	Ara-CSK_F	GCGCGCcatatgATGGTTGCTTCTGCTTCCG
		Ara-CSK_R	GCGCGCctcgagTGCTTCATTGGCTTCAGAT
**SDM**	Hik2^{C19S}	C19S_F	TTAATTTCCCTTTCTCAGTCACAGGTT
		C19S_R	AACCTGTGACTGAGAAAGGGAAATTAA
	Hik2^{C35S}	C35S_F	GGTGGACTGGTCCGGCGTTTATCTA
		C35S_R	TAGATAAACGCCGGACCAGTCCACC
	Hik2^{C153S}	C153S_F	TTAGCGGTGGCCTCCCTATTGGACCAA
		C153S_R	TTGGTCCAATAGGGAGGCCACCGCTAA
**qRT-PCR**	*PsaA*	PsaA-F	GGCACAAGCATCTCAGGTAA
		PsaA-R	AGCCCAAACAATGGATTCAA
	PsbA	PsbA-F	GGTTACAGATTCGGGCAAGA
		PsbA-R	AATACCTACTACCGGCCAAGC
	PsbD	PsbD-F	CTTTAGGGGGTTGGTTCACA
		PsbD-R	GCTTCAGGACCCCACAGTAA
	PetB	PetB-F	ATTGGGCGGTCAAAATTGTA
		PetB-R	AGACGGCCGTAAGAAGAGGT
	AtpB	AtpB-F	ATGAGTGCGACAGAGGGTTT
		AtpB-R	TGTGCGAGTATCGACAGGAC
	NdhC	NdhC-F	TCAAGTGCTATTCCTGTTTTGG
		NdhC-R	GAGCAAACATATAATAACGGATTCT
	RbcL	RbcL-F	CACCACAAACAGAGACTAAAGCA
		RbcL-R	CCTGCTTCTTCAGGTGGAAC
	Actin8	Actin8-F	TTCCAGCAGATGTGGATCTCTA
		Actin8-R	AGAAAGAAATGTGATCCCGTCA

Supplementary Table 3. Primer pairs used for cloning *Synechocystis* sp. PCC 6803, *Phaeodactylum tricornutum*, and *Arabidopsis thaliana CSK* coding sequences. Lowercase letters denote restriction sites. The serine triplet codon is highlighted in yellow.

Supplementary Methods

CSK-thylakoid interaction assay. Thylakoid membranes equivalent to 20 µg chlorophyll was spotted onto a PVDF membrane (GE Healthcare) by capillary action. The spotted membrane was allowed to fully dry in a fume hood before further processing. The membrane was blocked with 3 % Bovine Serum Albumin (BSA) in Tris Buffer Saline Tween 20 (TBST) buffer for 2 hours at room temperature. Immunopurified CSK-HA-FLAG protein was incubated with the PVDF membrane at 4 °C overnight, followed by washing twice in TBST buffer for 10 minutes each. The membrane was then incubated with anti-HA primary antibody (3F10, Roche) at a dilution of 1:1000 for 2 hours at room temperature, followed by incubation in horseradish peroxidase-coupled anti-rat secondary antibody (PI31470, Fisher Scientific) at a dilution of 1:2500 for 2 hours at room temperature. The membrane was then washed twice in TBST buffer for 10 minutes each. The immunoreactive signal was developed using a chemiluminescence detection reagent (Clarity Western ECL Substrate, Bio-Rad).

Sequence Analysis. Sequence similarity search was carried out with blastP and blastn using public databases Cyanobase (http://genome.kazusa.or.jp/cyanobase) and Joint Genome Institute (JGI) (http://www.jgi.doe.gov/). Domain prediction was carried out using the SMART database (http://smart.embl-heidelberg.de/smart/set_mode.cgi?NORMAL=1) (1). Amphipathicity of GAF domains CSK proteins were predicted using a web-based software: http://lbqp.unb.br/NetWheels/

Supplementary References

1. Schultz J, Milpetz F, Bork P, & Ponting CP (1998) SMART, a simple modular architecture research tool: identification of signaling domains. *Proc Natl Acad Sci U S A* 95(11):5857-5864.
