# Supplementary material for: An evolutionarily conserved iron-sulfur cluster underlies redox sensory function of the Chloroplast Sensor Kinase
Source: Commun Biol. 2020 Jan 8;3:13. doi: 10.1038/s42003-019-0728-4 (PMC6949291; doi:10.1038/s42003-019-0728-4)
Supplement: Supplementary file 2 — Description of Additional Supplementary Files [file 42003_2019_728_MOESM2_ESM.pdf]

**Descriptions of additional supplementary files**

**Supplementary data 1.** An excel file containing data points used to generate the following figures and tables

1. Data points used to plot UV-Vis absorbance spectra of Fig. 1
2. Temperature-dependent EPR traces as given in Fig. 2
3. Fig. 3 UV-Vis and EPR data
4. Fig. 4 EPR data and x-ray absorbance spectrum
5. Fig. 5 redox titration data based on EPR spin intensity
6. Size Exclusion Chromatography and far-UV-CD data points of Fig. 7
7. qPCR data of Fig. 8
8. Data points used to plot UV-Vis absorbance spectra of Supplementary Fig. 4
9. Size Exclusion Chromatography calibration data of Supplementary Fig. 7
10. Data points for Fe and S quantification as presented in Supplementary Table 1.
