# Supplementary material for: An evolutionarily conserved iron-sulfur cluster underlies redox sensory function of the Chloroplast Sensor Kinase
Source: Commun Biol. 2020 Jan 8;3:13. doi: 10.1038/s42003-019-0728-4 (PMC6949291; doi:10.1038/s42003-019-0728-4)
Supplement: Supplementary file 4 — Reporting Summary [file 42003_2019_728_MOESM4_ESM.pdf]

nature research

Corresponding author(s): Sujith Puthiyaveetil

Last updated by author(s): Dec 8, 2019

Reporting Summary

Nature Research wishes to improve the reproducibility of the work that we publish. This form provides structure for consistency and transparency in reporting. For further information on Nature Research policies, see Authors & Referees and the Editorial Policy Checklist.

Statistics

For all statistical analyses, confirm that the following items are present in the figure legend, table legend, main text, or Methods section.

n/a	Confirmed	
☐	☒	The exact sample size (n) for each experimental group/condition, given as a discrete number and unit of measurement
☐	☒	A statement on whether measurements were taken from distinct samples or whether the same sample was measured repeatedly
☐	☒	The statistical test(s) used AND whether they are one- or two-sided<br>*Only common tests should be described solely by name; describe more complex techniques in the Methods section.*
☒	☐	A description of all covariates tested
☒	☐	A description of any assumptions or corrections, such as tests of normality and adjustment for multiple comparisons
☐	☒	A full description of the statistical parameters including central tendency (e.g. means) or other basic estimates (e.g. regression coefficient) AND variation (e.g. standard deviation) or associated estimates of uncertainty (e.g. confidence intervals)
☐	☒	For null hypothesis testing, the test statistic (e.g. F, t, r) with confidence intervals, effect sizes, degrees of freedom and P value noted<br>*Give P values as exact values whenever suitable.*
☒	☐	For Bayesian analysis, information on the choice of priors and Markov chain Monte Carlo settings
☒	☐	For hierarchical and complex designs, identification of the appropriate level for tests and full reporting of outcomes
☒	☐	Estimates of effect sizes (e.g. Cohen's d, Pearson's r), indicating how they were calculated

Our web collection on statistics for biologists contains articles on many of the points above.

Software and code

Policy information about availability of computer code

Data collection	UV Solutions 4.2 (UV-Vis absorbance data); StepOne Software v2.3 (qPCR); CLARIOstar (plate reader); Image Lab (Western blot);Typhoon FLA 9500 Software (Autoradiograph); WINEPR (EPR); UNICORN 7.1 (FPLC and Size Exclusion Chromatography); Pro-Data Chirascan (CD spectroscopy); No custom-made software was used in the acquisition of data.
Data analysis	Excel and Sigmaplot (plotting and statistics); MARS (Plate reader); ClustalOmega (multiple sequence alignment); Jalview 2.11.0 (alignment editor); GraphPad Prism 6.01 (for fitting redox titration data and for generating the qRT-PCR plot); WINEPR (EPR data integration); Athena (EXAFS data processing); Artemis (EXAFS data curve fitting); OmniGraffle 7.12.1 (Image preparation); No custom-made data analysis codes were used in our study.

For manuscripts utilizing custom algorithms or software that are central to the research but not yet described in published literature, software must be made available to editors/reviewers. We strongly encourage code deposition in a community repository (e.g. GitHub). See the Nature Research guidelines for submitting code & software for further information.

Data

Policy information about availability of data

All manuscripts must include a data availability statement. This statement should provide the following information, where applicable:

- Accession codes, unique identifiers, or web links for publicly available datasets
- A list of figures that have associated raw data
- A description of any restrictions on data availability

The datasets generated during and/or analysed during the current study are available from the corresponding author on reasonable request.

Field-specific reporting

Please select the one below that is the best fit for your research. If you are not sure, read the appropriate sections before making your selection.

☒ Life sciences ☐ Behavioural & social sciences ☐ Ecological, evolutionary & environmental sciences

For a reference copy of the document with all sections, see nature.com/documents/nr-reporting-summary-flat.pdf

Life sciences study design

All studies must disclose on these points even when the disclosure is negative.

Sample size	Each measurement was done with 3-4 replicates and each experiment was performed at least 3-4 times.
Data exclusions	No data was excluded from the study.
Replication	3-4 replications per measurement
Randomization	Not relevant. We are working with protein samples.
Blinding	Not applicable because our measurements are biochemical and spectroscopic.

Reporting for specific materials, systems and methods

We require information from authors about some types of materials, experimental systems and methods used in many studies. Here, indicate whether each material, system or method listed is relevant to your study. If you are not sure if a list item applies to your research, read the appropriate section before selecting a response.

Materials & experimental systems

n/a	Involved in the study	
☐	☒	Antibodies
☒	☐	Eukaryotic cell lines
☒	☐	Palaeontology
☒	☐	Animals and other organisms
☒	☐	Human research participants
☒	☐	Clinical data

Methods

n/a	Involved in the study	
☒	☐	ChIP-seq
☒	☐	Flow cytometry
☒	☐	MRI-based neuroimaging

Antibodies

Antibodies used	Anti-HA and anti-FLAG antibodies
Validation	These antibodies come with the FLAG® HA Tandem Affinity Purification Kit (Sigma).
